# *Lactococcus lactis* Strain Plasma Uniquely Induces IFN-α Production via Plasmacytoid Dendritic Cell Activation: A Comparative Study of Postbiotic Products

**DOI:** 10.3390/microorganisms13102261

**Published:** 2025-09-26

**Authors:** Shigeru Fujimura, Masato Kawamura, Yurina Tamura

**Affiliations:** Division of Clinical Infectious Diseases & Chemotherapy, Graduate School of Pharmaceutical Sciences, Tohoku Medical and Pharmaceutical University, Sendai 981-8558, Japan; m-kawamura@tohoku-mpu.ac.jp (M.K.);

**Keywords:** *Lactococcus lactis* Plasma, interferon-α, plasmacytoid dendritic cells, postbiotics, antiviral immunity

## Abstract

Postbiotics are increasingly incorporated into functional foods and supplements due to their potential health benefits, particularly immune modulation. However, the mechanisms by which these products influence antiviral immunity remain incompletely understood. Type I interferons, especially interferon-α (IFN-α), are central mediators of early antiviral defense, acting primarily through the activation of plasmacytoid dendritic cells (pDCs). Five commercially available postbiotic products containing heat-killed bacterial strains were evaluated for their ability to stimulate pDCs and induce IFN-α production. Bacterial uptake by pDCs was analyzed using confocal microscopy with Z-stack imaging, and IFN-α levels were quantified by ELISA. Among the tested strains, only *Lactococcus lactis* strain Plasma (LC-Plasma) demonstrated significant internalization by pDCs and induced measurable IFN-α production (73.8 ± 2.5 pg/mL) at the recommended daily dose. This effect was not observed with other strains, even at higher bacterial loads (up to 1 × 10^11^ cells). Z-stack imaging confirmed that LC-Plasma was actively phagocytosed by pDCs, whereas other strains, such as *L. paracasei* MCC1849, adhered to the cell surface without internalization. The pDC concentration used in the assay approximated physiological levels in human blood. Notably, the IFN-α level induced by LC-Plasma exceeded that reported in the serum of hospitalized COVID-19 patients. *L. lactis* strain Plasma uniquely activates pDCs and induces IFN-α production under physiologically relevant conditions, distinguishing it from other postbiotic strains. These findings suggest that LC-Plasma may serve as a functional postbiotic with the potential to enhance antiviral immunity and mitigate disease severity.

## 1. Introduction

Influenza and other viral respiratory infections have caused numerous global outbreaks throughout history, including the Spanish flu, which resulted in significant morbidity and mortality [1,2]. More recently, the coronavirus disease 2019 (COVID-19) pandemic has had a substantial impact on healthcare systems and economies worldwide [3]. Viral infections remain a major public health concern due to their high transmissibility and the frequent emergence of novel variants, which contribute to reinfection and reduced vaccine efficacy [4]. Although vaccines and antiviral agents are currently used to prevent and manage viral infections [5], limitations such as adverse effects, high costs, and variable effectiveness have led to decreased acceptance in some populations, particularly in Japan [6]. Furthermore, conventional treatments primarily alleviate symptoms, whereas viral clearance depends on the host’s innate immune response. Type I interferons (IFNs), particularly interferon-α (IFN-α), play a critical role in the early defense against viral infections by promoting antiviral gene expression and limiting viral replication [7,8,9].

In recent years, increasing attention has been paid to functional foods and dietary supplements containing postbiotics for their potential immunostimulatory effects [10,11]. The concept of postbiotics has attracted considerable attention as an alternative or complement to probiotics. According to the International Scientific Association for Probiotics and Prebiotics (ISAPP), postbiotics are defined as “a preparation of inanimate microorganisms and/or their components that confers a health benefit on the host [12].” Unlike probiotics, which require viable microorganisms to exert their effects, postbiotics are derived from heat-killed bacterial cells or their structural components, such as peptidoglycans, lipoteichoic acids, or cell wall fragments, as well as microbial metabolites including short-chain fatty acids and bioactive peptides. Because postbiotics do not contain live bacteria, they offer important advantages in terms of safety, stability, and applicability in vulnerable populations, such as infants, the elderly, or immunocompromised patients. Accumulating evidence indicates that postbiotics can modulate host immune responses, enhance intestinal barrier function, and provide antimicrobial or antiviral effects. These properties suggest that postbiotics may represent a promising and safe strategy for functional food development and disease prevention. These products commonly include *Lactobacillus* spp. and other lactic acid bacteria, which are believed to activate mucosal immunity through interaction with Peyer’s patches and other gut-associated lymphoid tissues [13,14,15]. Despite their popularity, the clinical efficacy and underlying mechanisms of these products in antiviral immunity remain inadequately understood. In this study, we evaluated the immunomodulatory effects of bacterial components derived from commercially available postprobiotic products. Specifically, we focused on the activation of plasmacytoid dendritic cells (pDCs) and their ability to produce IFN-α, a key cytokine involved in antiviral immune responses. By comparing multiple strains under physiologically relevant conditions, we aimed to identify potential candidates that may contribute to host antiviral defense through functional dietary intervention.

## 2. Materials and Methods

### 2.1. Bacterial Strains

Heat-killed bacterial preparations classified as postbiotics were obtained from five commercially available health supplement products, each containing one of the following: *Lactococcus lactis* strain Plasma (Kirin Holdings, Tokyo, Japan), *Lactobacillus acidophilus* L-92 (Asahi Group Holdings, Tokyo, Japan), *Gluconacetobacter hansenii* GK-1 (Kewpie, Tokyo, Japan), *Lactiplantibacillus plantarum* L-137 (House Wellness Foods, Hyogo, Japan), and *Lacticaseibacillus paracasei* MCC1849 (Morinaga Milk Industry, Tokyo, Japan). Each supplement was suspended in 50 mL of phosphate-buffered saline (PBS; Fujifilm Wako Pure Chemical, Osaka, Japan) to match the daily intake indicated for each product, with all preparations standardized to a final concentration equivalent to 100 billion cells. The daily dosages used were as follows: *L. lactis* strain Plasma, 100 billion cells/day; *L. acidophilus* L-92, 20 billion cells/day; *G. hansenii* GK-1, 40 billion cells/day; *L. plantarum* L-137, 10 billion cells/day; and *L. paracasei* MCC1849, 50 billion cells/day.

### 2.2. 16S rRNA Gene Sequencing

Genomic DNA of each heat-killed strain was extracted using the InstaGene Matrix DNA Extraction Kit (Bio-Rad Laboratories, Hercules, CA, USA) according to the manufacturer’s instructions. The 16S rRNA gene was amplified by PCR using the universal primers 8UA and 1485B [16]. Nucleotide sequences were aligned with reference sequences for *L. lactis* ATCC19435 (GenBank accession no. AB008215.1), *L. acidophilus* ATCC4356 (AB008203.1), *G. hansenii* ATCC23769 (AB166734.1), *L. plantarum* NRRL B-14768 (NR_042394), and *L. paracasei* ATCC25302 (NR_117987) using BioEdit software 7.7.1. Sequence similarity was assessed using the BLAST+ 2.17.0 tool (https://blast.ncbi.nlm.nih.gov/Blast.cgi) to identify bacterial species. Strains were confirmed at the species level if sequence similarity with the reference strain was ≥98.7% [17].

### 2.3. FITC Staining of Bacterial Strains

Heat-killed strains were stained using a modified version of the procedure described in a previous study [18,19]. In short, each heat-killed strain prepared as described in the previous section was resuspended in 0.1 M sodium bicarbonate (NaHCO_3_). FITC isomer I (DOJINDO LABORATORIES, Kumamoto, Japan) was added to the bacterial suspension at a final concentration of 0.1 mg/mL. The mixture was vortexed and incubated at room temperature for 60 min in the dark. After incubation, the suspension was centrifuged at 3000 rpm for 10 min, and the supernatant was removed. The bacterial pellet was resuspended in 1 mL of sterile PBS and washed to remove any unstained FITC.

### 2.4. pDC Induction

Bone marrow fluid (HAMRI, Ibaraki, Japan) was obtained from four-week-old female BALB/cAnN CrlCrlj mice. Bone marrow-derived dendritic cells (BM-DCs) were cultured at a concentration of 1 × 10^6^ cells/mL in RPMI 1640 medium (Merck KGaA, Darmstadt, Germany) supplemented with 10% fetal bovine serum (FBS; Fujifilm Wako Pure Chemical, Osaka, Japan), 100 U/mL penicillin-streptomycin (Thermo Fisher Scientific, Tokyo, Japan), 1 mM sodium pyruvate (Thermo Fisher Scientific), 2.5 mM N-(2-hydroxyethyl)piperazine-N’-2-ethanesulfonic acid (HEPES; Thermo Fisher Scientific), 1% minimum essential medium non-essential amino acids (MEM-NEAA; Thermo Fisher Scientific), 50 μM 2-mercaptoethanol (2-ME; Thermo Fisher Scientific), and 100 ng/mL Fms-like tyrosine kinase 3 ligand (Flt3L; R&D Systems, Minneapolis, MN, USA). Cultures were maintained at 37 °C in a 5% CO_2_ atmosphere for 7 days [18]. The resulting pDCs were suspended in CellBanker 2 (Nippon Zenyaku Kogyo, Fukushima, Japan) at a concentration of 2 × 10^6^ viable cells/mL and stored at −80 °C.

### 2.5. pDC Phagocytosis Assay

BM-DCs were cryopreserved in CellBanker2 at –80 °C and subsequently thawed and washed with RPMI1640 medium (Merck KGaA, Darmstadt, Germany). pDCs were purified using the EasySep™ Mouse CD11b Positive Selection Kit II (Veritas Corporation, Tokyo, Japan). The purity of the isolated pDC population exceeded 95%. The phagocytosis assay was performed based on a previously reported method [19,20,21,22]. Micro cover glasses were placed in each well of a 24-well plate. Purified pDCs and FITC-stained bacteria were then added to the wells and co-cultured for 24 h at 37 °C in a 5% CO_2_ atmosphere. After incubation, the cover glasses were gently washed with PBS to remove non-adherent cells and bacteria, and the samples were fixed with 10% neutral-buffered formalin. The fixed samples were washed three times with PBS and then blocked with 1% bovine serum albumin (BSA) for 30 min to prevent non-specific antibody binding. Next, the cells were incubated with anti-mouse CD45R/B220 primary antibody (clone RA3-6B2; BioLegend, San Diego, CA, USA) at room temperature for 2 h.

CD45R/B220 is widely recognized as a pan-B cell marker; however, it is also well established that plasmacytoid dendritic cells (pDCs) in mice express this antigen. Therefore, anti-mouse CD45R/B220 antibody has been commonly used in combination with other pDC markers such as PDCA-1, Siglec-H, and low levels of CD11c to identify the pDC population [23]. In this study, CD45R/B220 was used as one of the markers to demonstrate pDCs involved in bacterial uptake. After washing, the cells were incubated in the dark with Alexa Fluor™ 546-conjugated goat anti-rat IgG (H+L), cross-adsorbed secondary antibody (Thermo Fisher Scientific, Waltham, MA, USA) for 30 min at room temperature. Samples were washed three times with PBS. Fluorescence images were acquired using a laser scanning confocal microscope (LSM900; Carl Zeiss, Oberkochen, Germany). Z-stack images were captured and processed using ZEN software version 3.01 (Carl Zeiss, Germany).

### 2.6. IFN-α Production from pDCs

Next, 2 µL samples containing heat-killed bacteria from each product, adjusted to the equivalent of 1 × 10^11^ cells, were added to 200 µL of pDCs at 2 × 10^5^ cells/mL. CpG ODN1585 (Thermo Fisher Scientific), an artificial ligand for Toll-like receptor 9 that was reported to produce IFN-α [24], was used as a positive control in this study. After incubation at 37 °C and 5% CO_2_ for 24 h, the concentration of IFN-α produced by pDC was measured by ELISA using the Verikine^TM^ Mouse Interferon Alpha ELISA kit (PBL Assay Science, Piscataway, NJ, USA) [20,25]. This measurement of IFN-α was repeated three times.

## 3. Results

### 3.1. Images from Laser Scanning Confocal Fluorescence Microscopy

**Figure 1:**
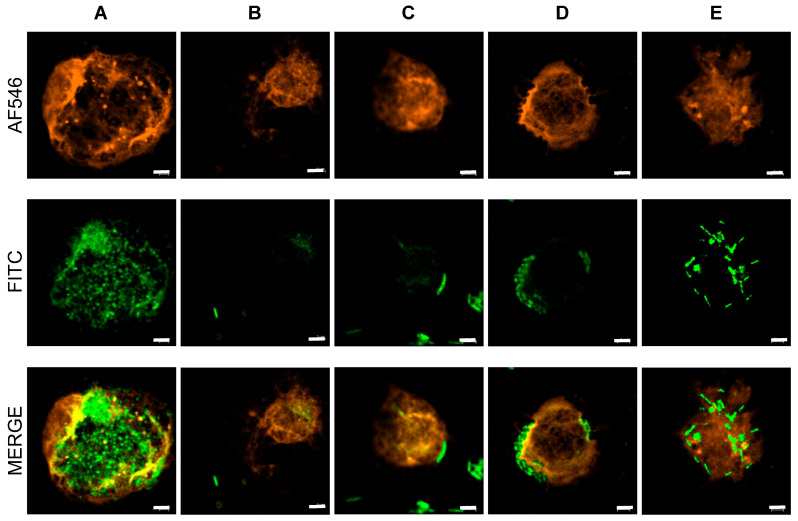
Confocal fluorescence microscopy images of plasmacytoid dendritic cells co-cultured with each heat-killed strain. pDCs were stained with Alexa Fluor™ 546-conjugated anti-mouse CD45R/B220 antibody (red), and bacterial strains were labeled with FITC (green). Co-localization of FITC-labeled bacteria with pDCs appears as yellow in merged images. Panels (**A**–**E**) correspond to the following strains: (**A**) Lactococcus lactis strain Plasma, (**B**) Lactobacillus acidophilus L-92, (**C**) Gluconacetobacter hansenii GK-1, (**D**) Lactiplantibacillus plantarum L-137, and (**E**) Lacticaseibacillus paracasei MCC1849. Co-localization was observed only in (**A**,**E**), indicating bacterial uptake by pDCs. Scale bar: 2 μm.

**Figure 2:**
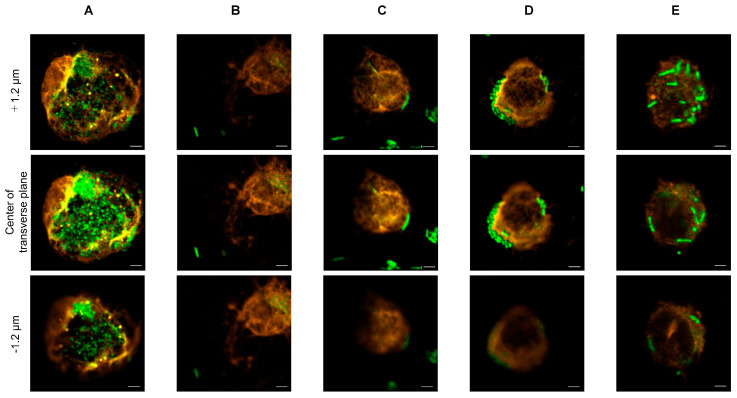
Z-axis confocal imaging of plasmacytoid dendritic cells co-cultured with each heat-killed strain. (**A**) Lactococcus lactis strain Plasma, (**B**) Lactbacillus acidophilus L-92, (**C**) Gluconacetobacter hansenii GK-1, (**D**) Lactiplantibacillus plantarum L-137, (**E**) Lacticaseibacillus paracasei MCC1849. Z-stack images were acquired to assess the spatial localization of FITC-labeled bacteria (green) relative to pDCs stained with Alexa Fluor™ 546-conjugated anti-mouse CD45R/B220 antibody (red). (Panel (**A**)) shows Lactococcus lactis strain Plasma localized within the central optical section of the pDC, indicating internalization. (Panel (**E**)) shows Lacticaseibacillus paracasei MCC1849 localized approximately +1.2 μm above the central plane of the pDC, suggesting surface association or partial uptake. (Panels (**B**–**D**)) did not demonstrate clear evidence of bacterial internalization. Scale bar: 2 μm.

To evaluate phagocytic activity, FITC-stained bacteria and pDCs stained with Alexa Fluor 546 were visualized, as shown in Figure 1. In the merged images, *L. lactis* strain Plasma and *L. paracasei* MCC1849 appeared co-localized with pDCs, as indicated by the overlapping fluorescence signals (Figure 1A,E). In contrast, no evidence of phagocytosis by pDCs was observed with the other bacterial strains (Figure 1B–D). Furthermore, Z-stack images (optical sections through the sample) revealed that numerous *L. lactis* strain Plasma cells were localized within the central optical plane of pDCs (Figure 2A), suggesting internalization. In contrast, *L. paracasei* MCC1849 cells appeared co-localized at approximately +1.2 µm above the central plane of pDCs (Figure 2E), suggesting possible surface association rather than full internalization.

### 3.2. IFN-α Production by pDCs Induced by Each Strain

**Figure 3:**
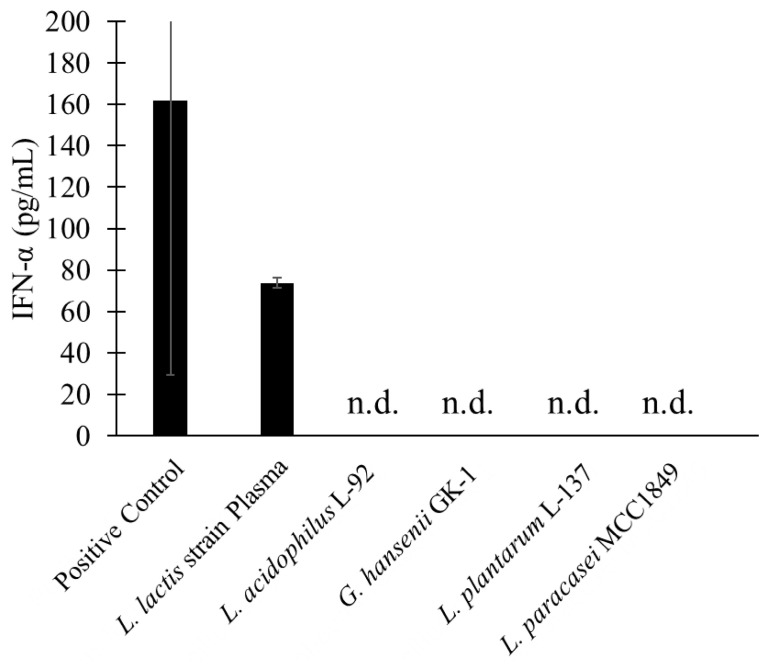
Comparison of IFN-α production from plasmacytoid dendritic cells induced by each heat-killed strain. The five heat-killed strains were standardized to 1 × 10^11^ cells and co-cultured with pDCs for 24 h. Only the product containing Lactococcus lactis strain Plasma induced measurable IFN-α production. Data are presented as mean ± standard deviation (SD).

The product containing *L. lactis* strain Plasma induced IFN-α production under conditions equivalent to the recommended daily intake (1 × 10^11^ cells), yielding a concentration of 73.83 ± 2.53 pg/mL (Figure 3). IFN-α production remained below the detection limit for *L. acidophilus* L-92, *G. hansenii* GK-1, *L. plantarum* L-137, and *L. paracasei* MCC1849.

## 4. Discussion

Postbiotics refer to the oral intake of heat-killed bacteria, such as *L. lactis* strain Plasma, with the aim of maintaining health [12]. Live probiotic bacteria were excluded from the phagocytosis assay using pDCs because, under normal physiological conditions, orally ingested live bacteria do not enter the bloodstream. The presence of live bacteria in circulation would indicate bacteremia, a pathological condition. Therefore, it is biologically implausible for circulating dendritic cells to come into direct contact with viable probiotics. Previous reports have indicated that components derived from postbiotic bacteria can directly activate dendritic cells and enhance immune responses [20]. pDCs represent a unique subset of dendritic cells that specialize in the production of type I IFN [26]. The production of type I IFN is part of the earliest responses to viral infection and acts on the infected cells to increase the expression of antiviral IFN response genes. This response limits viral replication and suppresses the spread of infection at the tissue level [27].

In this study, we investigated the immunomodulatory effects of various heat-killed strains on pDCs, with a specific focus on their capacity to induce IFN-α, a key cytokine in antiviral immune responses. Among the tested strains, only *L. lactis* strain Plasma demonstrated a significant capacity to be phagocytosed by pDCs and to stimulate IFN-α production. Confocal fluorescence microscopy revealed co-localization of *L. lactis* strain Plasma and *L. paracasei* MCC1849 with pDCs, suggesting physical interaction. However, Z-stack analysis further clarified that only *L. lactis* strain Plasma was internalized by pDCs, as evidenced by its presence in the central optical plane of the cells. This indicates active phagocytosis, whereas *L. paracasei* MCC1849 may have only adhered to the cell surface. Among all tested strains, only *L. lactis* strain Plasma induced detectable IFN-α levels (73.83 ± 2.53 pg/mL) at the recommended daily intake concentration. Notably, even when the bacterial load was increased to 100 billion cells for the other strains, no IFN-α production was observed. This suggests that the ability to stimulate IFN-α is not solely dependent on bacterial quantity, but rather on specific molecular components or structural properties unique to *L. lactis* strain Plasma. Recent literature indicates that peptidoglycan structures are preferentially recognized by phagocytic cells, including dendritic cells, through mechanisms requiring phagocytosis [28,29]. Moreover, *Lactococcus lactis* has been shown to induce IFN-α via TLR9- and MyD88-dependent pathways [20], consistent with the role of microbial DNA sensing by pDCs [30]. Reviews on PRRs further confirm that bacterial cell wall architecture influences host immunostimulation [29,31].

In the IFN-α production assay conducted in this study, pDCs constituted 30.9% of the total cell population. Accordingly, the pDC concentration in the cell suspension (2 × 10^5^ cells/mL) was estimated to be 6.18 × 10^4^ cells/mL. According to previous reports by Lichtner et al. [32] and Moss et al. [33], approximately 1 × 10^4^ pDCs/mL are present in human peripheral blood. Thus, the pDC concentration used in this study approximates physiological levels in humans.

Type I interferons, particularly IFN-α, are rapidly induced during the early phase of many viral infections, playing a critical role in restricting viral replication and shaping adaptive immunity [34]. For example, influenza virus infection is typically associated with a robust induction of IFN-α by plasmacytoid dendritic cells, which contributes to efficient viral clearance [35]. In contrast, several viruses have evolved mechanisms to suppress or evade type I interferon responses. Notably, severe cases of COVID-19 are characterized by impaired IFN-α production, despite high viral loads, reflecting viral strategies that antagonize innate immune signaling pathways [8]. This divergent pattern indicates that IFN-α responses vary not only by the type of virus but also by the stage and severity of infection. Contoli et al. showed that blood IFN-α levels in hospitalized COVID-19 patients were significantly lower compared to controls [11 (6–34) vs. 42 (24–87) pg/mL, *p* < 0.01] [36]. In the present study, stimulation of pDCs by *Lactococcus lactis* strain Plasma resulted in IFN-α production at a level of 73.8 pg/mL. Therefore, modulation of IFN-α induction by microbial components, such as postbiotics, may represent an important avenue for enhancing antiviral immunity, particularly in settings where endogenous interferon responses are inadequate. These findings suggest that the postbiotic properties of *L. lactis* strain Plasma may aid in preventing early-stage viral infections and potentially mitigate disease severity in hospitalized patients.

This study has the limitation that heat-killed versions of conventional probiotics were not examined. Inclusion of these strains would have provided valuable information regarding whether the immunostimulatory activity observed is unique to *Lactococcus lactis* strain Plasma or a broader feature of heat-killed probiotics. Future investigations addressing this point will help clarify the mechanisms through which postbiotics modulate antiviral immunity.

## 5. Conclusions

Given the critical role of IFN-α in antiviral defense, *L. lactis* strain Plasma appears to be a promising functional postbiotic for enhancing innate immunity, particularly against respiratory viral infections [37,38,39]. Further mechanistic studies are warranted to elucidate the specific molecular patterns responsible for pDC activation and to evaluate the clinical efficacy of *L. lactis* strain Plasma in human subjects.

## Data Availability

The original contributions presented in this study are included in the article. Further inquiries can be directed to the corresponding author.
